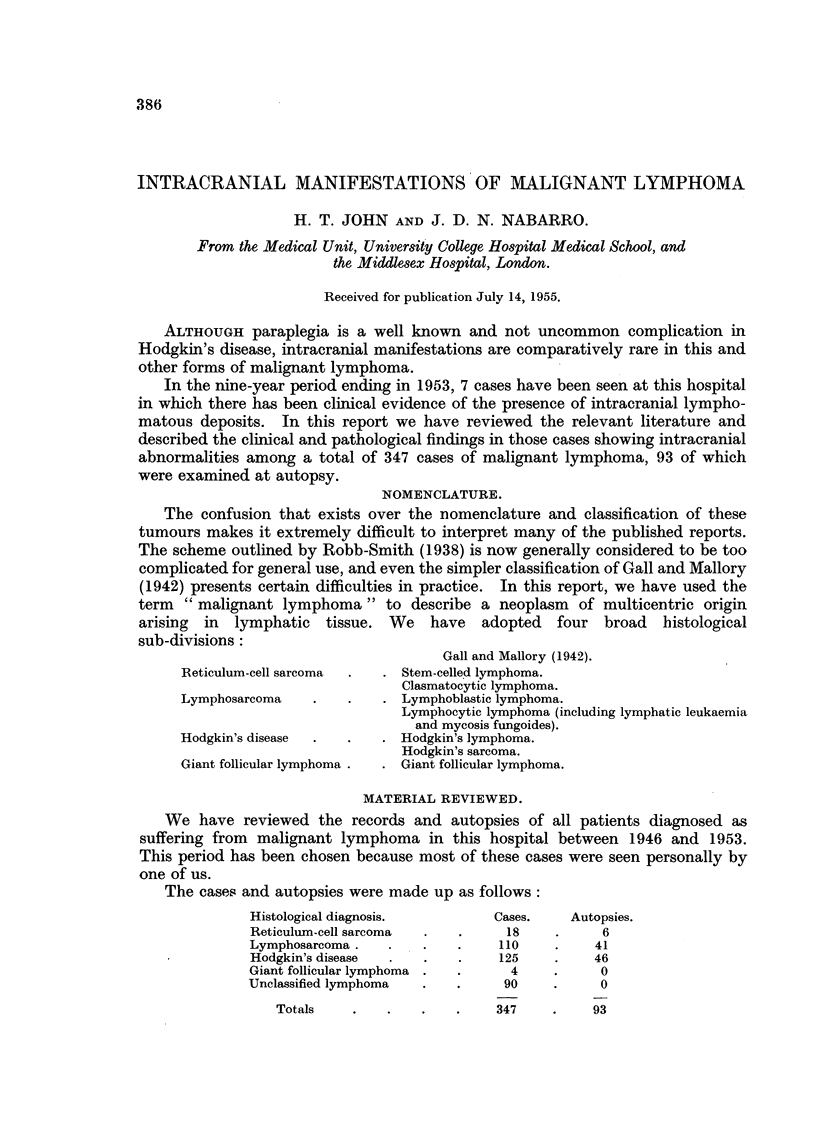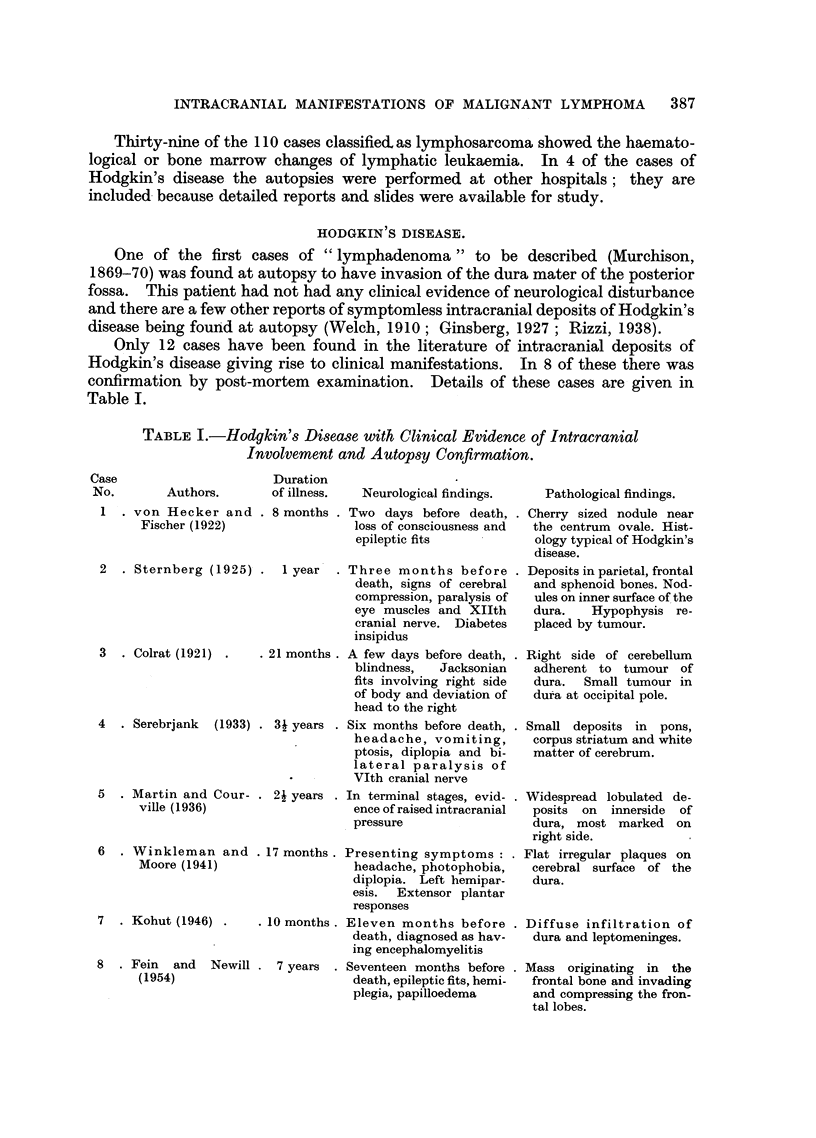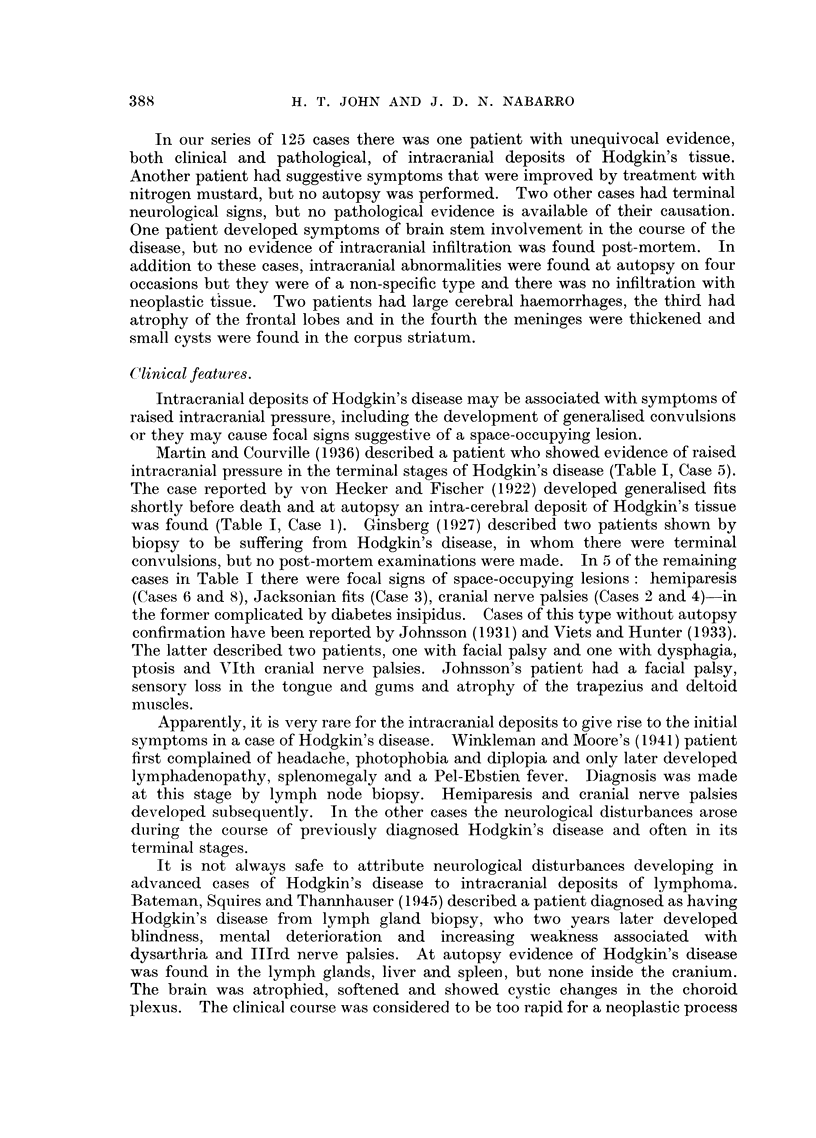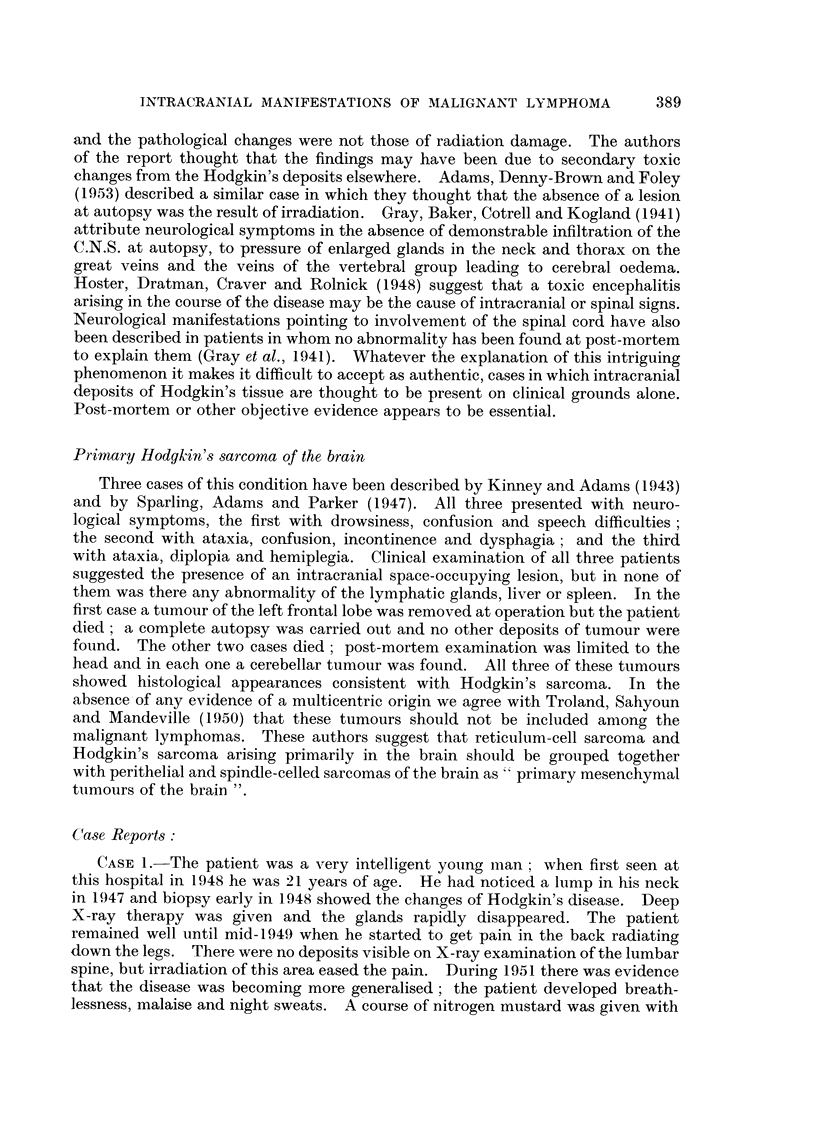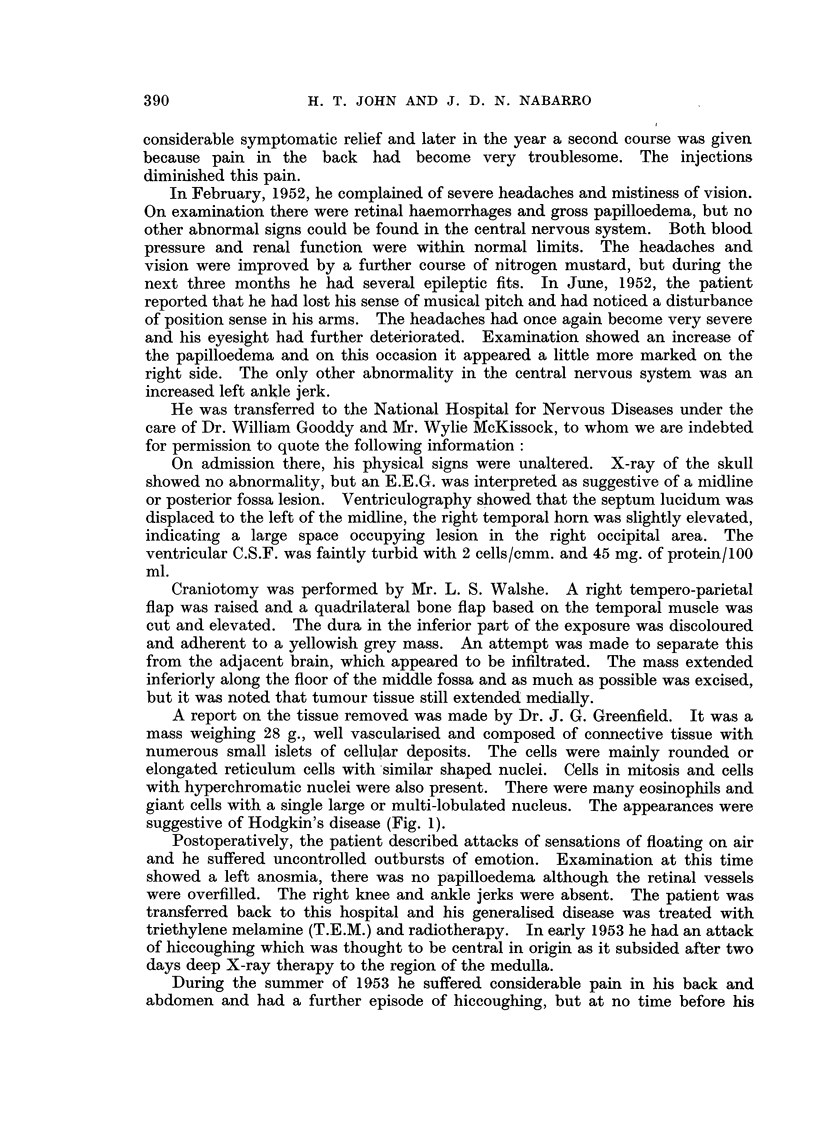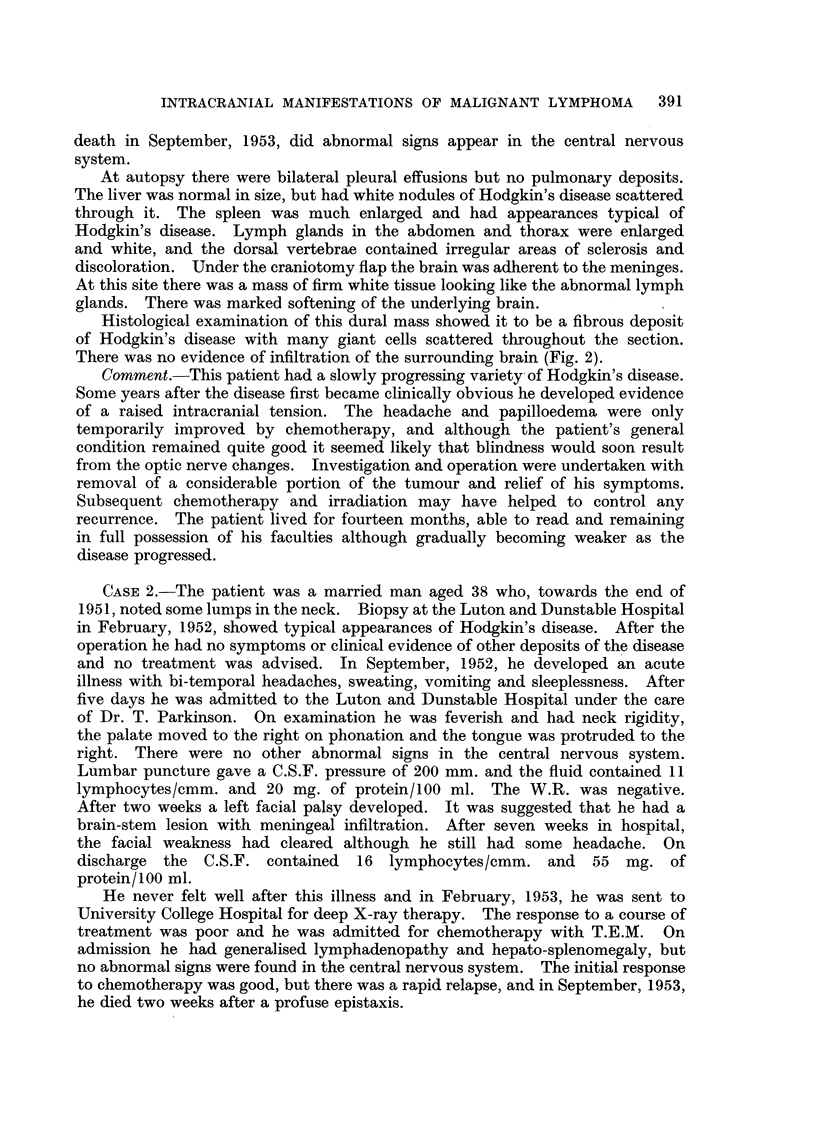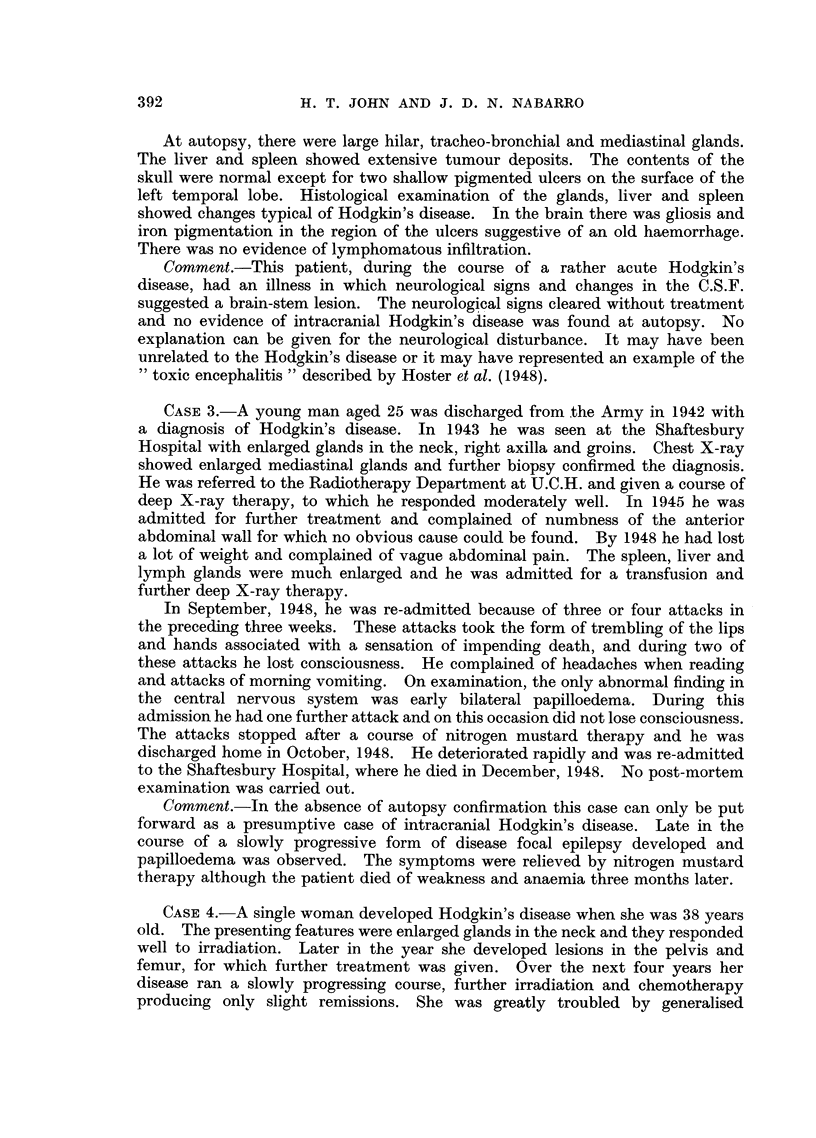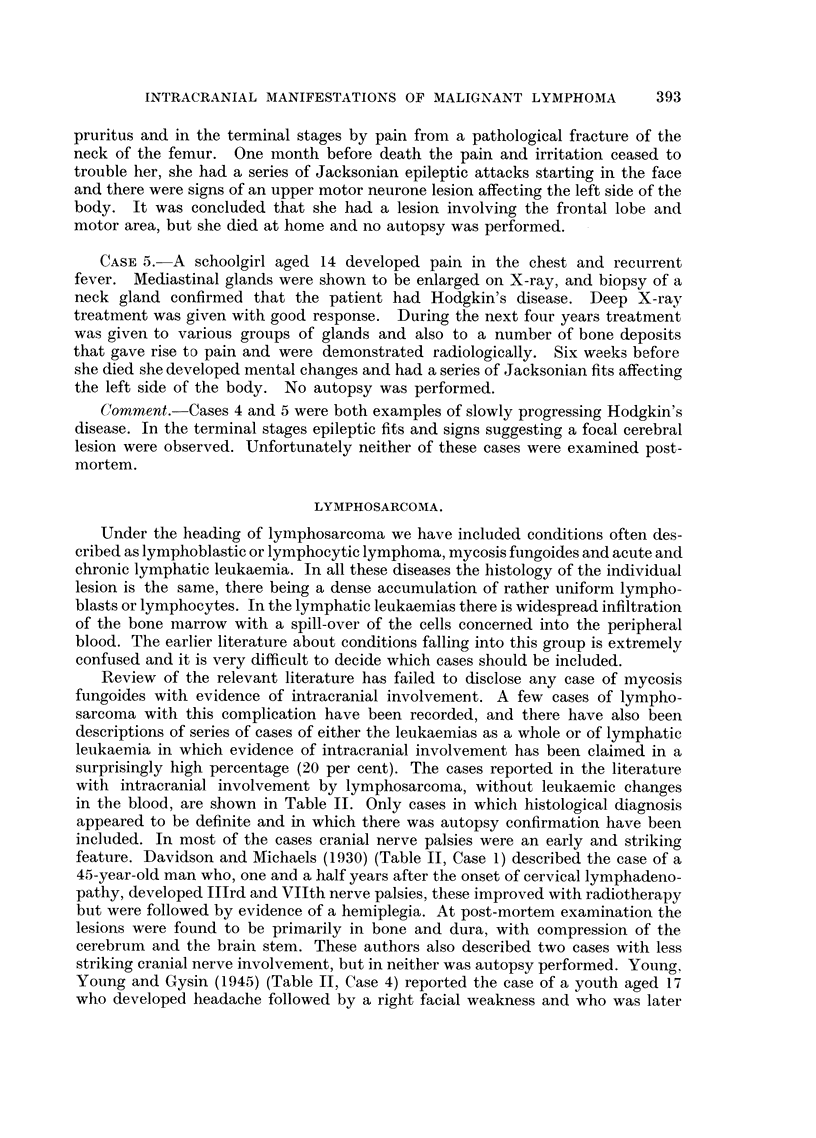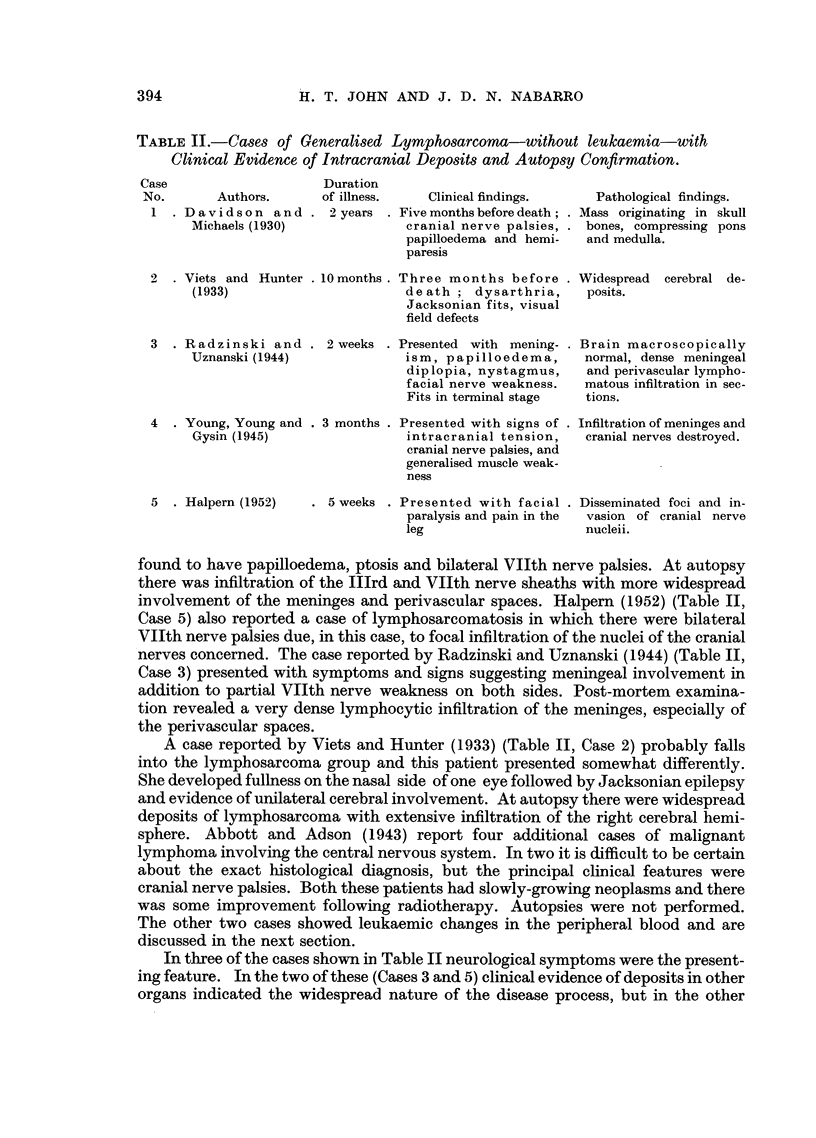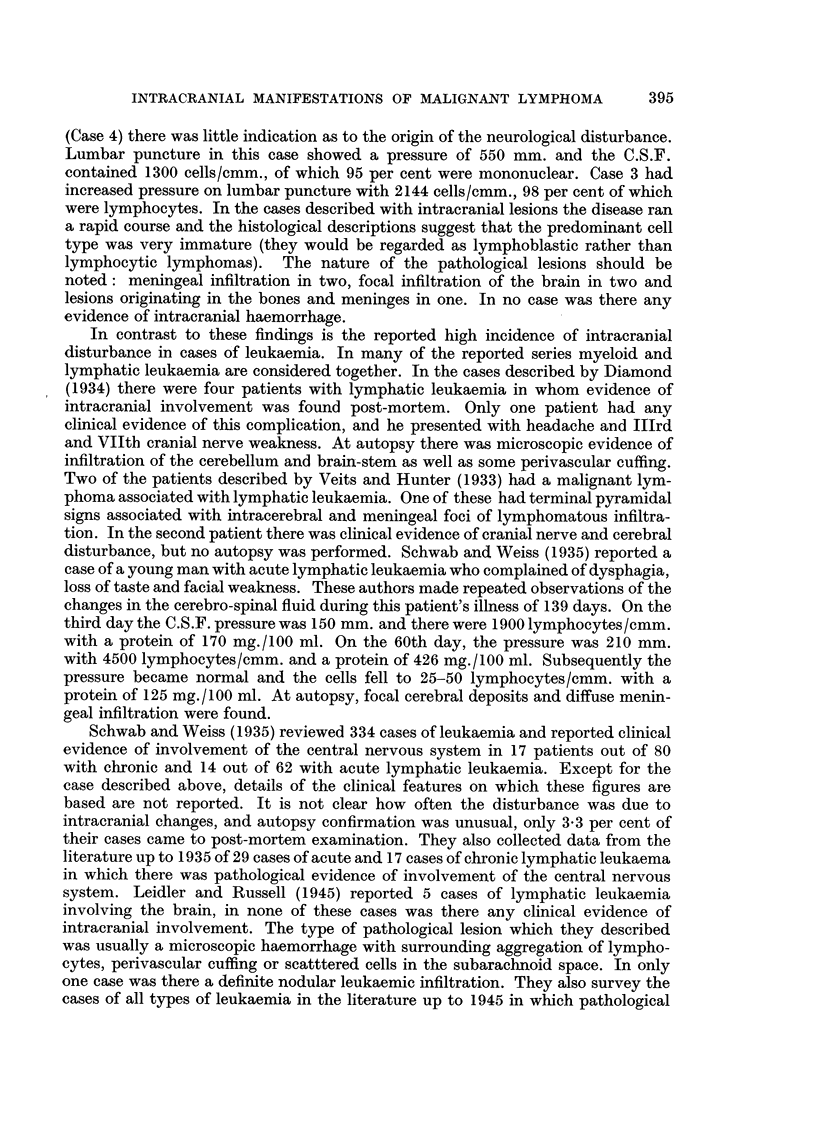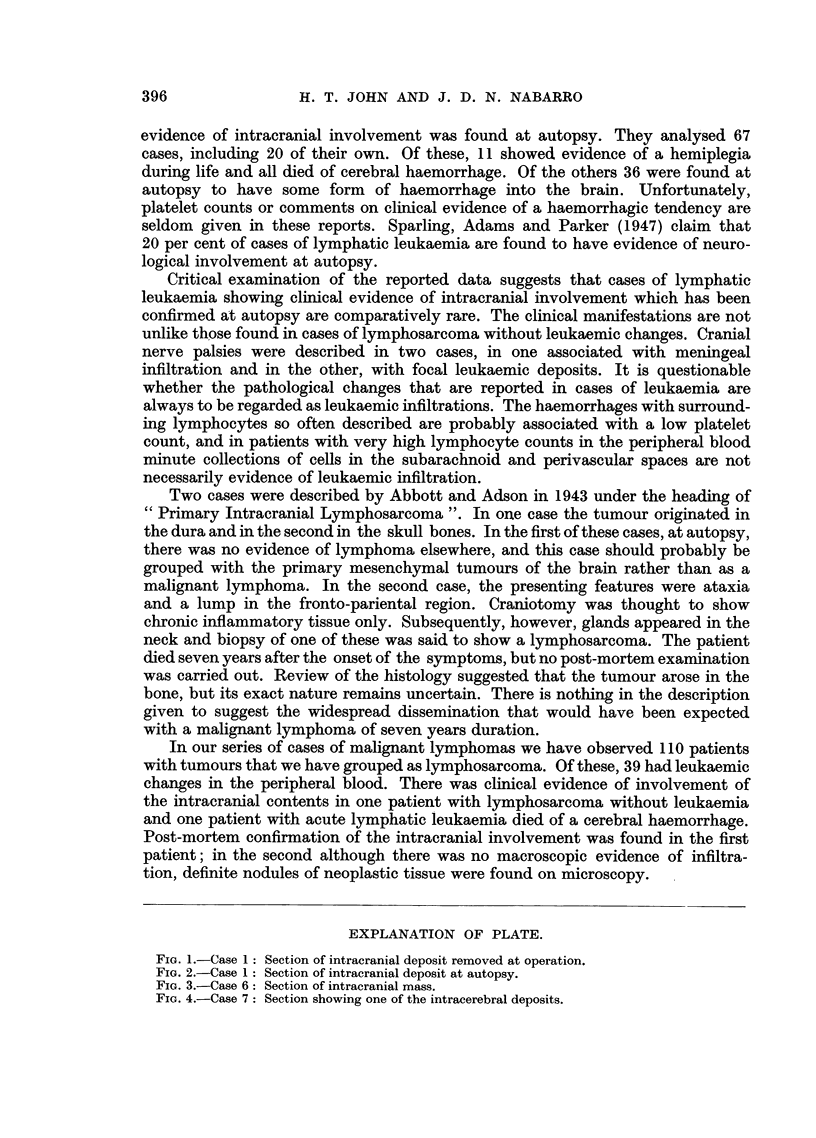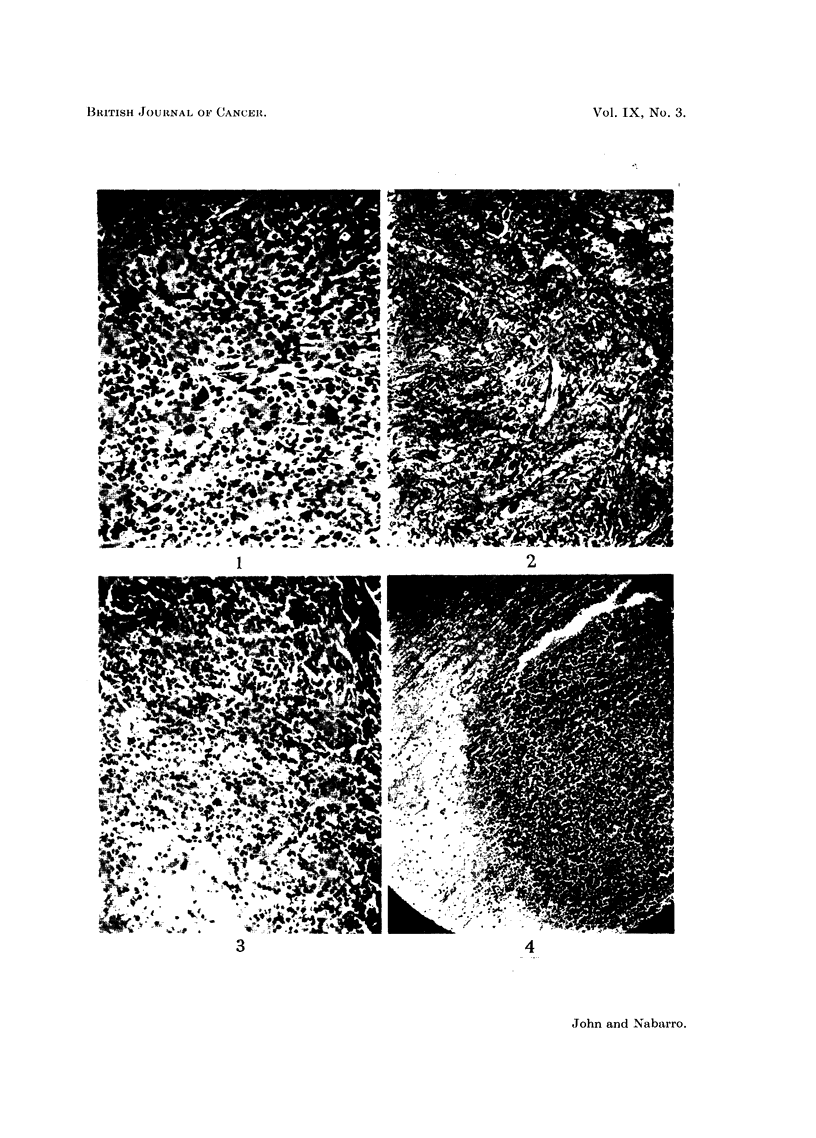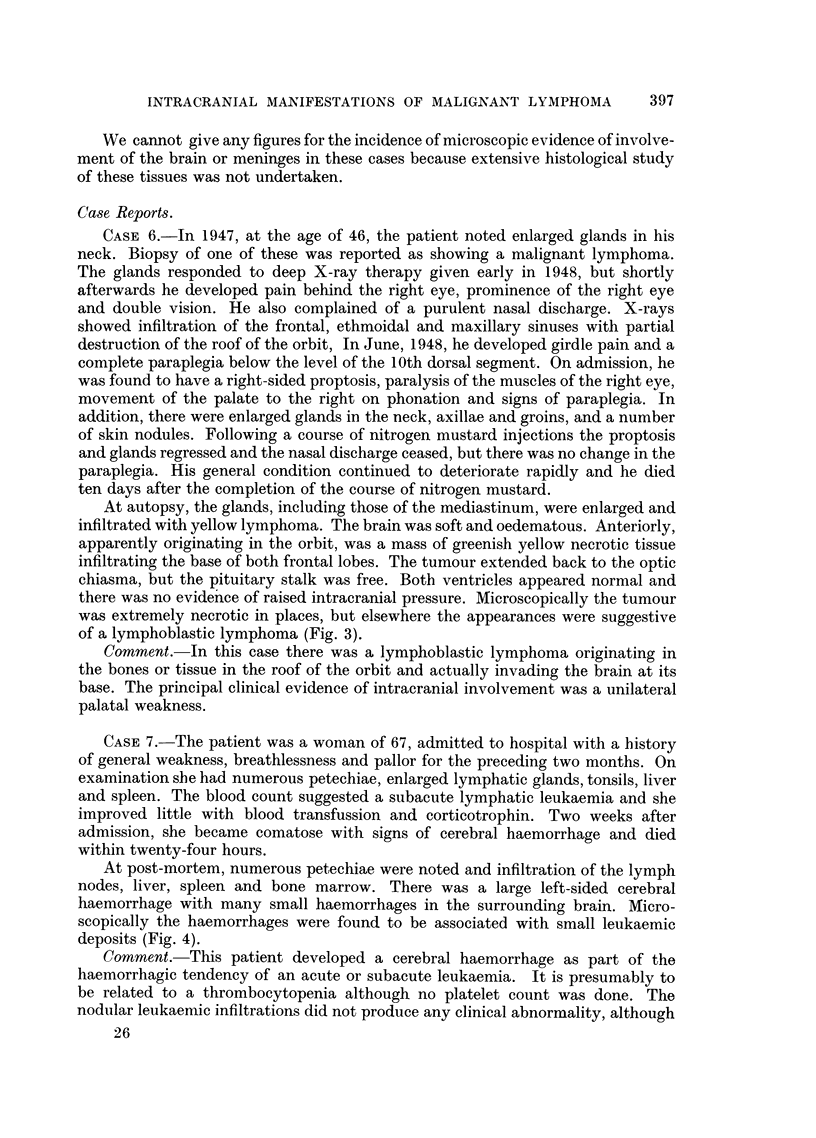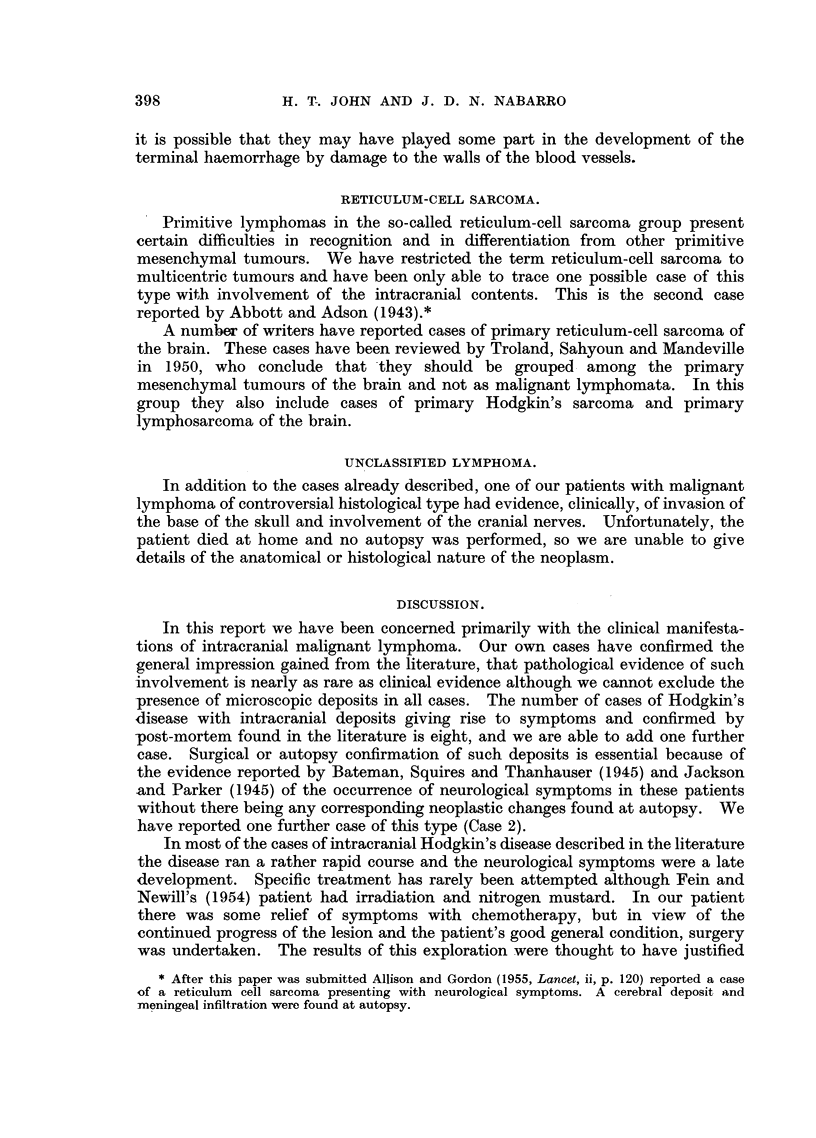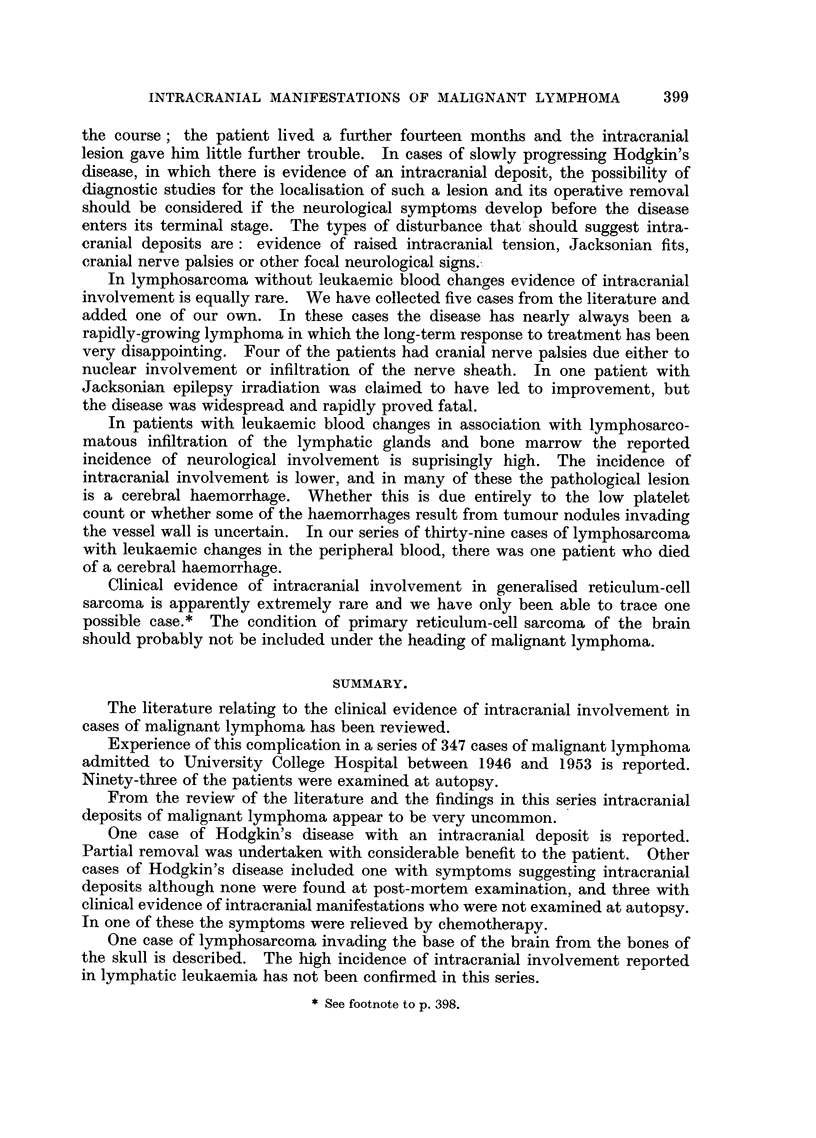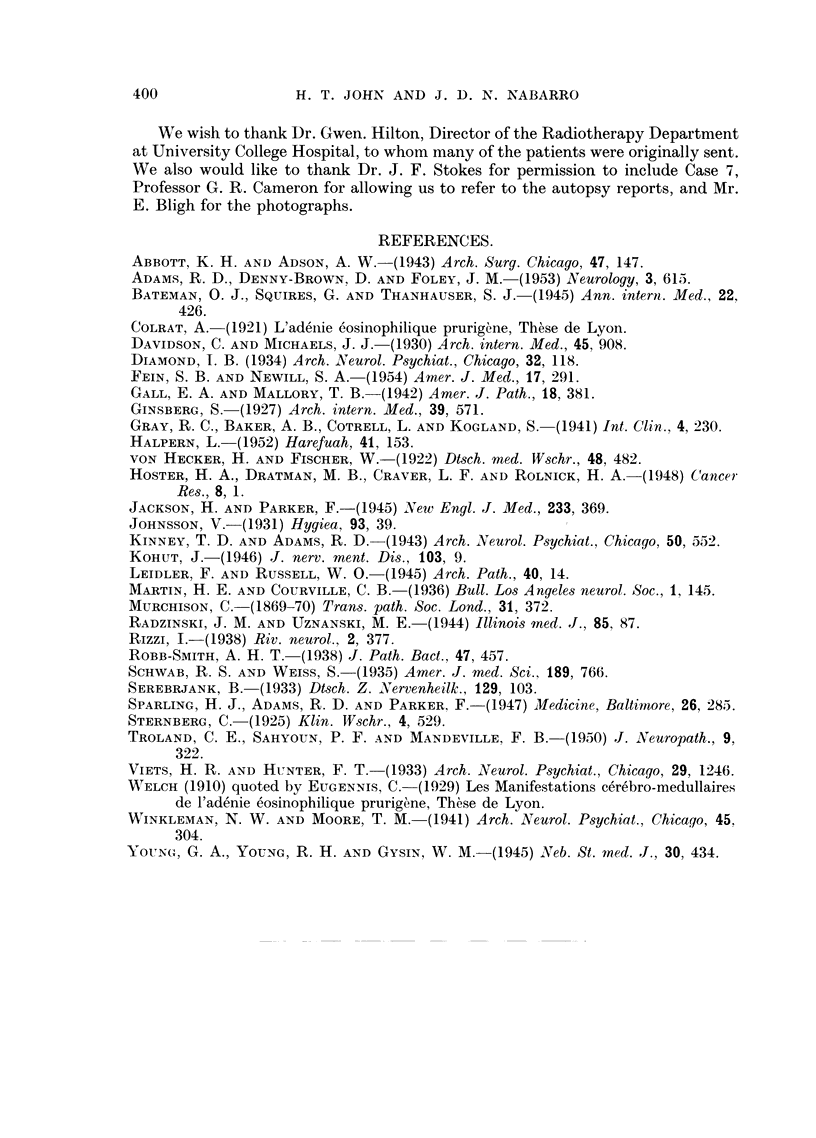# Intracranial Manifestations of Malignant Lymphoma

**DOI:** 10.1038/bjc.1955.37

**Published:** 1955-09

**Authors:** H. T. John, J. D. N. Nabarro

## Abstract

**Images:**


### INTRACRANIAL MANIFESTATIONS OF MALIGNANT LYMPHOMA

H. T. JOHN AND J. D. N. NABARRO.

*From the Medical Unit, University College Hospital Medical School, and the Middlesex Hospital, London.*

Received for publication July 14, 1955.

ALTHOUGH paraplegia is a well known and not uncommon complication in Hodgkin's disease, intracranial manifestations are comparatively rare in this and other forms of malignant lymphoma.

In the nine-year period ending in 1953, 7 cases have been seen at this hospital in which there has been clinical evidence of the presence of intracranial lymphomatous deposits. In this report we have reviewed the relevant literature and described the clinical and pathological findings in those cases showing intracranial abnormalities among a total of 347 cases of malignant lymphoma, 93 of which were examined at autopsy.

### NOMENCLATURE.

The confusion that exists over the nomenclature and classification of these tumours makes it extremely difficult to interpret many of the published reports. The scheme outlined by Robb-Smith (1938) is now generally considered to be too complicated for general use, and even the simpler classification of Gall and Mallory (1942) presents certain difficulties in practice. In this report, we have used the term " malignant lymphoma " to describe a neoplasm of multicentric origin arising in lymphatic tissue. We have adopted four broad histological sub-divisions :

| | Gall and Mallory (1942). |
|---|---|
| Reticulum-cell sarcoma | Stem-celled lymphoma.<br>Clasmatocytic lymphoma. |
| Lymphosarcoma | Lymphoblastic lymphoma.<br>Lymphocytic lymphoma (including lymphatic leukaemia and mycosis fungoides). |
| Hodgkin's disease | Hodgkin's lymphoma.<br>Hodgkin's sarcoma. |
| Giant follicular lymphoma | Giant follicular lymphoma. |

### MATERIAL REVIEWED.

We have reviewed the records and autopsies of all patients diagnosed as suffering from malignant lymphoma in this hospital between 1946 and 1953. This period has been chosen because most of these cases were seen personally by one of us.

The cases and autopsies were made up as follows :

| Histological diagnosis. | Cases. | Autopsies. |
|---|---|---|
| Reticulum-cell sarcoma | 18 | 6 |
| Lymphosarcoma | 110 | 41 |
| Hodgkin's disease | 125 | 46 |
| Giant follicular lymphoma | 4 | 0 |
| Unclassified lymphoma | 90 | 0 |
| Totals | 347 | 93 |

Thirty-nine of the 110 cases classified as lymphosarcoma showed the haematological or bone marrow changes of lymphatic leukaemia. In 4 of the cases of Hodgkin's disease the autopsies were performed at other hospitals; they are included because detailed reports and slides were available for study.

### HODGKIN'S DISEASE.

One of the first cases of "lymphadenoma" to be described (Murchison, 1869–70) was found at autopsy to have invasion of the dura mater of the posterior fossa. This patient had not had any clinical evidence of neurological disturbance and there are a few other reports of symptomless intracranial deposits of Hodgkin's disease being found at autopsy (Welch, 1910; Ginsberg, 1927; Rizzi, 1938).

Only 12 cases have been found in the literature of intracranial deposits of Hodgkin's disease giving rise to clinical manifestations. In 8 of these there was confirmation by post-mortem examination. Details of these cases are given in Table I.

TABLE I.—*Hodgkin's Disease with Clinical Evidence of Intracranial Involvement and Autopsy Confirmation.*

| Case No. | Authors. | Duration of illness. | Neurological findings. | Pathological findings. |
|---|---|---|---|---|
| 1 | von Hecker and Fischer (1922) | 8 months | Two days before death, loss of consciousness and epileptic fits | Cherry sized nodule near the centrum ovale. Histology typical of Hodgkin's disease. |
| 2 | Sternberg (1925) | 1 year | Three months before death, signs of cerebral compression, paralysis of eye muscles and XIIth cranial nerve. Diabetes insipidus | Deposits in parietal, frontal and sphenoid bones. Nodules on inner surface of the dura. Hypophysis replaced by tumour. |
| 3 | Colrat (1921) | 21 months | A few days before death, blindness, Jacksonian fits involving right side of body and deviation of head to the right | Right side of cerebellum adherent to tumour of dura. Small tumour in dura at occipital pole. |
| 4 | Serebrjank (1933) | $3\frac{1}{2}$ years | Six months before death, headache, vomiting, ptosis, diplopia and bilateral paralysis of VIth cranial nerve | Small deposits in pons, corpus striatum and white matter of cerebrum. |
| 5 | Martin and Courville (1936) | $2\frac{1}{2}$ years | In terminal stages, evidence of raised intracranial pressure | Widespread lobulated deposits on innerside of dura, most marked on right side. |
| 6 | Winkleman and Moore (1941) | 17 months | Presenting symptoms: headache, photophobia, diplopia. Left hemiparesis. Extensor plantar responses | Flat irregular plaques on cerebral surface of the dura. |
| 7 | Kohut (1946) | 10 months | Eleven months before death, diagnosed as having encephalomyelitis | Diffuse infiltration of dura and leptomeninges. |
| 8 | Fein and Newill (1954) | 7 years | Seventeen months before death, epileptic fits, hemiplegia, papilloedema | Mass originating in the frontal bone and invading and compressing the frontal lobes. |

In our series of 125 cases there was one patient with unequivocal evidence, both clinical and pathological, of intracranial deposits of Hodgkin's tissue. Another patient had suggestive symptoms that were improved by treatment with nitrogen mustard, but no autopsy was performed. Two other cases had terminal neurological signs, but no pathological evidence is available of their causation. One patient developed symptoms of brain stem involvement in the course of the disease, but no evidence of intracranial infiltration was found post-mortem. In addition to these cases, intracranial abnormalities were found at autopsy on four occasions but they were of a non-specific type and there was no infiltration with neoplastic tissue. Two patients had large cerebral haemorrhages, the third had atrophy of the frontal lobes and in the fourth the meninges were thickened and small cysts were found in the corpus striatum.

*Clinical features.*

Intracranial deposits of Hodgkin's disease may be associated with symptoms of raised intracranial pressure, including the development of generalised convulsions or they may cause focal signs suggestive of a space-occupying lesion.

Martin and Courville (1936) described a patient who showed evidence of raised intracranial pressure in the terminal stages of Hodgkin's disease (Table I, Case 5). The case reported by von Hecker and Fischer (1922) developed generalised fits shortly before death and at autopsy an intra-cerebral deposit of Hodgkin's tissue was found (Table I, Case 1). Ginsberg (1927) described two patients shown by biopsy to be suffering from Hodgkin's disease, in whom there were terminal convulsions, but no post-mortem examinations were made. In 5 of the remaining cases in Table I there were focal signs of space-occupying lesions : hemiparesis (Cases 6 and 8), Jacksonian fits (Case 3), cranial nerve palsies (Cases 2 and 4)—in the former complicated by diabetes insipidus. Cases of this type without autopsy confirmation have been reported by Johnsson (1931) and Viets and Hunter (1933). The latter described two patients, one with facial palsy and one with dysphagia, ptosis and VIth cranial nerve palsies. Johnsson's patient had a facial palsy, sensory loss in the tongue and gums and atrophy of the trapezius and deltoid muscles.

Apparently, it is very rare for the intracranial deposits to give rise to the initial symptoms in a case of Hodgkin's disease. Winkleman and Moore's (1941) patient first complained of headache, photophobia and diplopia and only later developed lymphadenopathy, splenomegaly and a Pel-Ebstien fever. Diagnosis was made at this stage by lymph node biopsy. Hemiparesis and cranial nerve palsies developed subsequently. In the other cases the neurological disturbances arose during the course of previously diagnosed Hodgkin's disease and often in its terminal stages.

It is not always safe to attribute neurological disturbances developing in advanced cases of Hodgkin's disease to intracranial deposits of lymphoma. Bateman, Squires and Thannhauser (1945) described a patient diagnosed as having Hodgkin's disease from lymph gland biopsy, who two years later developed blindness, mental deterioration and increasing weakness associated with dysarthria and IIIrd nerve palsies. At autopsy evidence of Hodgkin's disease was found in the lymph glands, liver and spleen, but none inside the cranium. The brain was atrophied, softened and showed cystic changes in the choroid plexus. The clinical course was considered to be too rapid for a neoplastic process and the pathological changes were not those of radiation damage. The authors of the report thought that the findings may have been due to secondary toxic changes from the Hodgkin's deposits elsewhere. Adams, Denny-Brown and Foley (1953) described a similar case in which they thought that the absence of a lesion at autopsy was the result of irradiation. Gray, Baker, Cotrell and Kogland (1941) attribute neurological symptoms in the absence of demonstrable infiltration of the C.N.S. at autopsy, to pressure of enlarged glands in the neck and thorax on the great veins and the veins of the vertebral group leading to cerebral oedema. Hoster, Dratman, Craver and Rolnick (1948) suggest that a toxic encephalitis arising in the course of the disease may be the cause of intracranial or spinal signs. Neurological manifestations pointing to involvement of the spinal cord have also been described in patients in whom no abnormality has been found at post-mortem to explain them (Gray *et al.*, 1941). Whatever the explanation of this intriguing phenomenon it makes it difficult to accept as authentic, cases in which intracranial deposits of Hodgkin's tissue are thought to be present on clinical grounds alone. Post-mortem or other objective evidence appears to be essential.

*Primary Hodgkin's sarcoma of the brain*

Three cases of this condition have been described by Kinney and Adams (1943) and by Sparling, Adams and Parker (1947). All three presented with neurological symptoms, the first with drowsiness, confusion and speech difficulties ; the second with ataxia, confusion, incontinence and dysphagia ; and the third with ataxia, diplopia and hemiplegia. Clinical examination of all three patients suggested the presence of an intracranial space-occupying lesion, but in none of them was there any abnormality of the lymphatic glands, liver or spleen. In the first case a tumour of the left frontal lobe was removed at operation but the patient died ; a complete autopsy was carried out and no other deposits of tumour were found. The other two cases died ; post-mortem examination was limited to the head and in each one a cerebellar tumour was found. All three of these tumours showed histological appearances consistent with Hodgkin's sarcoma. In the absence of any evidence of a multicentric origin we agree with Troland, Sahyoun and Mandeville (1950) that these tumours should not be included among the malignant lymphomas. These authors suggest that reticulum-cell sarcoma and Hodgkin's sarcoma arising primarily in the brain should be grouped together with perithelial and spindle-celled sarcomas of the brain as " primary mesenchymal tumours of the brain ".

*Case Reports :*

CASE 1.—The patient was a very intelligent young man ; when first seen at this hospital in 1948 he was 21 years of age. He had noticed a lump in his neck in 1947 and biopsy early in 1948 showed the changes of Hodgkin's disease. Deep X-ray therapy was given and the glands rapidly disappeared. The patient remained well until mid-1949 when he started to get pain in the back radiating down the legs. There were no deposits visible on X-ray examination of the lumbar spine, but irradiation of this area eased the pain. During 1951 there was evidence that the disease was becoming more generalised ; the patient developed breathlessness, malaise and night sweats. A course of nitrogen mustard was given with considerable symptomatic relief and later in the year a second course was given because pain in the back had become very troublesome. The injections diminished this pain.

In February, 1952, he complained of severe headaches and mistiness of vision. On examination there were retinal haemorrhages and gross papilloedema, but no other abnormal signs could be found in the central nervous system. Both blood pressure and renal function were within normal limits. The headaches and vision were improved by a further course of nitrogen mustard, but during the next three months he had several epileptic fits. In June, 1952, the patient reported that he had lost his sense of musical pitch and had noticed a disturbance of position sense in his arms. The headaches had once again become very severe and his eyesight had further deteriorated. Examination showed an increase of the papilloedema and on this occasion it appeared a little more marked on the right side. The only other abnormality in the central nervous system was an increased left ankle jerk.

He was transferred to the National Hospital for Nervous Diseases under the care of Dr. William Gooddy and Mr. Wylie McKissock, to whom we are indebted for permission to quote the following information :

On admission there, his physical signs were unaltered. X-ray of the skull showed no abnormality, but an E.E.G. was interpreted as suggestive of a midline or posterior fossa lesion. Ventriculography showed that the septum lucidum was displaced to the left of the midline, the right temporal horn was slightly elevated, indicating a large space occupying lesion in the right occipital area. The ventricular C.S.F. was faintly turbid with 2 cells/cmm. and 45 mg. of protein/100 ml.

Craniotomy was performed by Mr. L. S. Walshe. A right tempero-parietal flap was raised and a quadrilateral bone flap based on the temporal muscle was cut and elevated. The dura in the inferior part of the exposure was discoloured and adherent to a yellowish grey mass. An attempt was made to separate this from the adjacent brain, which appeared to be infiltrated. The mass extended inferiorly along the floor of the middle fossa and as much as possible was excised, but it was noted that tumour tissue still extended medially.

A report on the tissue removed was made by Dr. J. G. Greenfield. It was a mass weighing 28 g., well vascularised and composed of connective tissue with numerous small islets of cellular deposits. The cells were mainly rounded or elongated reticulum cells with similar shaped nuclei. Cells in mitosis and cells with hyperchromatic nuclei were also present. There were many eosinophils and giant cells with a single large or multi-lobulated nucleus. The appearances were suggestive of Hodgkin's disease (Fig. 1).

Postoperatively, the patient described attacks of sensations of floating on air and he suffered uncontrolled outbursts of emotion. Examination at this time showed a left anosmia, there was no papilloedema although the retinal vessels were overfilled. The right knee and ankle jerks were absent. The patient was transferred back to this hospital and his generalised disease was treated with triethylene melamine (T.E.M.) and radiotherapy. In early 1953 he had an attack of hiccoughing which was thought to be central in origin as it subsided after two days deep X-ray therapy to the region of the medulla.

During the summer of 1953 he suffered considerable pain in his back and abdomen and had a further episode of hiccoughing, but at no time before his death in September, 1953, did abnormal signs appear in the central nervous system.

At autopsy there were bilateral pleural effusions but no pulmonary deposits. The liver was normal in size, but had white nodules of Hodgkin's disease scattered through it. The spleen was much enlarged and had appearances typical of Hodgkin's disease. Lymph glands in the abdomen and thorax were enlarged and white, and the dorsal vertebrae contained irregular areas of sclerosis and discoloration. Under the craniotomy flap the brain was adherent to the meninges. At this site there was a mass of firm white tissue looking like the abnormal lymph glands. There was marked softening of the underlying brain.

Histological examination of this dural mass showed it to be a fibrous deposit of Hodgkin's disease with many giant cells scattered throughout the section. There was no evidence of infiltration of the surrounding brain (Fig. 2).

*Comment.*—This patient had a slowly progressing variety of Hodgkin's disease. Some years after the disease first became clinically obvious he developed evidence of a raised intracranial tension. The headache and papilloedema were only temporarily improved by chemotherapy, and although the patient's general condition remained quite good it seemed likely that blindness would soon result from the optic nerve changes. Investigation and operation were undertaken with removal of a considerable portion of the tumour and relief of his symptoms. Subsequent chemotherapy and irradiation may have helped to control any recurrence. The patient lived for fourteen months, able to read and remaining in full possession of his faculties although gradually becoming weaker as the disease progressed.

CASE 2.—The patient was a married man aged 38 who, towards the end of 1951, noted some lumps in the neck. Biopsy at the Luton and Dunstable Hospital in February, 1952, showed typical appearances of Hodgkin's disease. After the operation he had no symptoms or clinical evidence of other deposits of the disease and no treatment was advised. In September, 1952, he developed an acute illness with bi-temporal headaches, sweating, vomiting and sleeplessness. After five days he was admitted to the Luton and Dunstable Hospital under the care of Dr. T. Parkinson. On examination he was feverish and had neck rigidity, the palate moved to the right on phonation and the tongue was protruded to the right. There were no other abnormal signs in the central nervous system. Lumbar puncture gave a C.S.F. pressure of 200 mm. and the fluid contained 11 lymphocytes/cmm. and 20 mg. of protein/100 ml. The W.R. was negative. After two weeks a left facial palsy developed. It was suggested that he had a brain-stem lesion with meningeal infiltration. After seven weeks in hospital, the facial weakness had cleared although he still had some headache. On discharge the C.S.F. contained 16 lymphocytes/cmm. and 55 mg. of protein/100 ml.

He never felt well after this illness and in February, 1953, he was sent to University College Hospital for deep X-ray therapy. The response to a course of treatment was poor and he was admitted for chemotherapy with T.E.M. On admission he had generalised lymphadenopathy and hepato-splenomegaly, but no abnormal signs were found in the central nervous system. The initial response to chemotherapy was good, but there was a rapid relapse, and in September, 1953, he died two weeks after a profuse epistaxis.

At autopsy, there were large hilar, tracheo-bronchial and mediastinal glands. The liver and spleen showed extensive tumour deposits. The contents of the skull were normal except for two shallow pigmented ulcers on the surface of the left temporal lobe. Histological examination of the glands, liver and spleen showed changes typical of Hodgkin's disease. In the brain there was gliosis and iron pigmentation in the region of the ulcers suggestive of an old haemorrhage. There was no evidence of lymphomatous infiltration.

*Comment.*—This patient, during the course of a rather acute Hodgkin's disease, had an illness in which neurological signs and changes in the C.S.F. suggested a brain-stem lesion. The neurological signs cleared without treatment and no evidence of intracranial Hodgkin's disease was found at autopsy. No explanation can be given for the neurological disturbance. It may have been unrelated to the Hodgkin's disease or it may have represented an example of the " toxic encephalitis " described by Hoster *et al.* (1948).

CASE 3.—A young man aged 25 was discharged from the Army in 1942 with a diagnosis of Hodgkin's disease. In 1943 he was seen at the Shaftesbury Hospital with enlarged glands in the neck, right axilla and groins. Chest X-ray showed enlarged mediastinal glands and further biopsy confirmed the diagnosis. He was referred to the Radiotherapy Department at U.C.H. and given a course of deep X-ray therapy, to which he responded moderately well. In 1945 he was admitted for further treatment and complained of numbness of the anterior abdominal wall for which no obvious cause could be found. By 1948 he had lost a lot of weight and complained of vague abdominal pain. The spleen, liver and lymph glands were much enlarged and he was admitted for a transfusion and further deep X-ray therapy.

In September, 1948, he was re-admitted because of three or four attacks in the preceding three weeks. These attacks took the form of trembling of the lips and hands associated with a sensation of impending death, and during two of these attacks he lost consciousness. He complained of headaches when reading and attacks of morning vomiting. On examination, the only abnormal finding in the central nervous system was early bilateral papilloedema. During this admission he had one further attack and on this occasion did not lose consciousness. The attacks stopped after a course of nitrogen mustard therapy and he was discharged home in October, 1948. He deteriorated rapidly and was re-admitted to the Shaftesbury Hospital, where he died in December, 1948. No post-mortem examination was carried out.

*Comment.*—In the absence of autopsy confirmation this case can only be put forward as a presumptive case of intracranial Hodgkin's disease. Late in the course of a slowly progressive form of disease focal epilepsy developed and papilloedema was observed. The symptoms were relieved by nitrogen mustard therapy although the patient died of weakness and anaemia three months later.

CASE 4.—A single woman developed Hodgkin's disease when she was 38 years old. The presenting features were enlarged glands in the neck and they responded well to irradiation. Later in the year she developed lesions in the pelvis and femur, for which further treatment was given. Over the next four years her disease ran a slowly progressing course, further irradiation and chemotherapy producing only slight remissions. She was greatly troubled by generalised pruritus and in the terminal stages by pain from a pathological fracture of the neck of the femur. One month before death the pain and irritation ceased to trouble her, she had a series of Jacksonian epileptic attacks starting in the face and there were signs of an upper motor neurone lesion affecting the left side of the body. It was concluded that she had a lesion involving the frontal lobe and motor area, but she died at home and no autopsy was performed.

CASE 5.—A schoolgirl aged 14 developed pain in the chest and recurrent fever. Mediastinal glands were shown to be enlarged on X-ray, and biopsy of a neck gland confirmed that the patient had Hodgkin's disease. Deep X-ray treatment was given with good response. During the next four years treatment was given to various groups of glands and also to a number of bone deposits that gave rise to pain and were demonstrated radiologically. Six weeks before she died she developed mental changes and had a series of Jacksonian fits affecting the left side of the body. No autopsy was performed.

*Comment.*—Cases 4 and 5 were both examples of slowly progressing Hodgkin's disease. In the terminal stages epileptic fits and signs suggesting a focal cerebral lesion were observed. Unfortunately neither of these cases were examined post-mortem.

### LYMPHOSARCOMA.

Under the heading of lymphosarcoma we have included conditions often described as lymphoblastic or lymphocytic lymphoma, mycosis fungoides and acute and chronic lymphatic leukaemia. In all these diseases the histology of the individual lesion is the same, there being a dense accumulation of rather uniform lymphoblasts or lymphocytes. In the lymphatic leukaemias there is widespread infiltration of the bone marrow with a spill-over of the cells concerned into the peripheral blood. The earlier literature about conditions falling into this group is extremely confused and it is very difficult to decide which cases should be included.

Review of the relevant literature has failed to disclose any case of mycosis fungoides with evidence of intracranial involvement. A few cases of lymphosarcoma with this complication have been recorded, and there have also been descriptions of series of cases of either the leukaemias as a whole or of lymphatic leukaemia in which evidence of intracranial involvement has been claimed in a surprisingly high percentage (20 per cent). The cases reported in the literature with intracranial involvement by lymphosarcoma, without leukaemic changes in the blood, are shown in Table II. Only cases in which histological diagnosis appeared to be definite and in which there was autopsy confirmation have been included. In most of the cases cranial nerve palsies were an early and striking feature. Davidson and Michaels (1930) (Table II, Case 1) described the case of a 45-year-old man who, one and a half years after the onset of cervical lymphadenopathy, developed IIIrd and VIIth nerve palsies, these improved with radiotherapy but were followed by evidence of a hemiplegia. At post-mortem examination the lesions were found to be primarily in bone and dura, with compression of the cerebrum and the brain stem. These authors also described two cases with less striking cranial nerve involvement, but in neither was autopsy performed. Young, Young and Gysin (1945) (Table II, Case 4) reported the case of a youth aged 17 who developed headache followed by a right facial weakness and who was later

TABLE II.—*Cases of Generalised Lymphosarcoma—without leukaemia—with Clinical Evidence of Intracranial Deposits and Autopsy Confirmation.*

| Case No. | Authors. | Duration of illness. | Clinical findings. | Pathological findings. |
|---|---|---|---|---|
| 1 | Davidson and Michaels (1930) | 2 years | Five months before death; cranial nerve palsies, papilloedema and hemiparesis | Mass originating in skull bones, compressing pons and medulla. |
| 2 | Viets and Hunter (1933) | 10 months | Three months before death; dysarthria, Jacksonian fits, visual field defects | Widespread cerebral deposits. |
| 3 | Radzinski and Uznanski (1944) | 2 weeks | Presented with meningism, papilloedema, diplopia, nystagmus, facial nerve weakness. Fits in terminal stage | Brain macroscopically normal, dense meningeal and perivascular lymphomatous infiltration in sections. |
| 4 | Young, Young and Gysin (1945) | 3 months | Presented with signs of intracranial tension, cranial nerve palsies, and generalised muscle weakness | Infiltration of meninges and cranial nerves destroyed. |
| 5 | Halpern (1952) | 5 weeks | Presented with facial paralysis and pain in the leg | Disseminated foci and invasion of cranial nerve nucleii. |

found to have papilloedema, ptosis and bilateral VIIth nerve palsies. At autopsy there was infiltration of the IIIrd and VIIth nerve sheaths with more widespread involvement of the meninges and perivascular spaces. Halpern (1952) (Table II, Case 5) also reported a case of lymphosarcomatosis in which there were bilateral VIIth nerve palsies due, in this case, to focal infiltration of the nuclei of the cranial nerves concerned. The case reported by Radzinski and Uznanski (1944) (Table II, Case 3) presented with symptoms and signs suggesting meningeal involvement in addition to partial VIIth nerve weakness on both sides. Post-mortem examination revealed a very dense lymphocytic infiltration of the meninges, especially of the perivascular spaces.

A case reported by Viets and Hunter (1933) (Table II, Case 2) probably falls into the lymphosarcoma group and this patient presented somewhat differently. She developed fullness on the nasal side of one eye followed by Jacksonian epilepsy and evidence of unilateral cerebral involvement. At autopsy there were widespread deposits of lymphosarcoma with extensive infiltration of the right cerebral hemisphere. Abbott and Adson (1943) report four additional cases of malignant lymphoma involving the central nervous system. In two it is difficult to be certain about the exact histological diagnosis, but the principal clinical features were cranial nerve palsies. Both these patients had slowly-growing neoplasms and there was some improvement following radiotherapy. Autopsies were not performed. The other two cases showed leukaemic changes in the peripheral blood and are discussed in the next section.

In three of the cases shown in Table II neurological symptoms were the presenting feature. In the two of these (Cases 3 and 5) clinical evidence of deposits in other organs indicated the widespread nature of the disease process, but in the other

(Case 4) there was little indication as to the origin of the neurological disturbance. Lumbar puncture in this case showed a pressure of 550 mm. and the C.S.F. contained 1300 cells/cmm., of which 95 per cent were mononuclear. Case 3 had increased pressure on lumbar puncture with 2144 cells/cmm., 98 per cent of which were lymphocytes. In the cases described with intracranial lesions the disease ran a rapid course and the histological descriptions suggest that the predominant cell type was very immature (they would be regarded as lymphoblastic rather than lymphocytic lymphomas). The nature of the pathological lesions should be noted : meningeal infiltration in two, focal infiltration of the brain in two and lesions originating in the bones and meninges in one. In no case was there any evidence of intracranial haemorrhage.

In contrast to these findings is the reported high incidence of intracranial disturbance in cases of leukaemia. In many of the reported series myeloid and lymphatic leukaemia are considered together. In the cases described by Diamond (1934) there were four patients with lymphatic leukaemia in whom evidence of intracranial involvement was found post-mortem. Only one patient had any clinical evidence of this complication, and he presented with headache and IIIrd and VIIth cranial nerve weakness. At autopsy there was microscopic evidence of infiltration of the cerebellum and brain-stem as well as some perivascular cuffing. Two of the patients described by Veits and Hunter (1933) had a malignant lymphoma associated with lymphatic leukaemia. One of these had terminal pyramidal signs associated with intracerebral and meningeal foci of lymphomatous infiltration. In the second patient there was clinical evidence of cranial nerve and cerebral disturbance, but no autopsy was performed. Schwab and Weiss (1935) reported a case of a young man with acute lymphatic leukaemia who complained of dysphagia, loss of taste and facial weakness. These authors made repeated observations of the changes in the cerebro-spinal fluid during this patient's illness of 139 days. On the third day the C.S.F. pressure was 150 mm. and there were 1900 lymphocytes/cmm. with a protein of 170 mg./100 ml. On the 60th day, the pressure was 210 mm. with 4500 lymphocytes/cmm. and a protein of 426 mg./100 ml. Subsequently the pressure became normal and the cells fell to 25–50 lymphocytes/cmm. with a protein of 125 mg./100 ml. At autopsy, focal cerebral deposits and diffuse meningeal infiltration were found.

Schwab and Weiss (1935) reviewed 334 cases of leukaemia and reported clinical evidence of involvement of the central nervous system in 17 patients out of 80 with chronic and 14 out of 62 with acute lymphatic leukaemia. Except for the case described above, details of the clinical features on which these figures are based are not reported. It is not clear how often the disturbance was due to intracranial changes, and autopsy confirmation was unusual, only 3·3 per cent of their cases came to post-mortem examination. They also collected data from the literature up to 1935 of 29 cases of acute and 17 cases of chronic lymphatic leukaema in which there was pathological evidence of involvement of the central nervous system. Leidler and Russell (1945) reported 5 cases of lymphatic leukaemia involving the brain, in none of these cases was there any clinical evidence of intracranial involvement. The type of pathological lesion which they described was usually a microscopic haemorrhage with surrounding aggregation of lymphocytes, perivascular cuffing or scatttered cells in the subarachnoid space. In only one case was there a definite nodular leukaemic infiltration. They also survey the cases of all types of leukaemia in the literature up to 1945 in which pathological evidence of intracranial involvement was found at autopsy. They analysed 67 cases, including 20 of their own. Of these, 11 showed evidence of a hemiplegia during life and all died of cerebral haemorrhage. Of the others 36 were found at autopsy to have some form of haemorrhage into the brain. Unfortunately, platelet counts or comments on clinical evidence of a haemorrhagic tendency are seldom given in these reports. Sparling, Adams and Parker (1947) claim that 20 per cent of cases of lymphatic leukaemia are found to have evidence of neurological involvement at autopsy.

Critical examination of the reported data suggests that cases of lymphatic leukaemia showing clinical evidence of intracranial involvement which has been confirmed at autopsy are comparatively rare. The clinical manifestations are not unlike those found in cases of lymphosarcoma without leukaemic changes. Cranial nerve palsies were described in two cases, in one associated with meningeal infiltration and in the other, with focal leukaemic deposits. It is questionable whether the pathological changes that are reported in cases of leukaemia are always to be regarded as leukaemic infiltrations. The haemorrhages with surrounding lymphocytes so often described are probably associated with a low platelet count, and in patients with very high lymphocyte counts in the peripheral blood minute collections of cells in the subarachnoid and perivascular spaces are not necessarily evidence of leukaemic infiltration.

Two cases were described by Abbott and Adson in 1943 under the heading of " Primary Intracranial Lymphosarcoma ". In one case the tumour originated in the dura and in the second in the skull bones. In the first of these cases, at autopsy, there was no evidence of lymphoma elsewhere, and this case should probably be grouped with the primary mesenchymal tumours of the brain rather than as a malignant lymphoma. In the second case, the presenting features were ataxia and a lump in the fronto-pariental region. Craniotomy was thought to show chronic inflammatory tissue only. Subsequently, however, glands appeared in the neck and biopsy of one of these was said to show a lymphosarcoma. The patient died seven years after the onset of the symptoms, but no post-mortem examination was carried out. Review of the histology suggested that the tumour arose in the bone, but its exact nature remains uncertain. There is nothing in the description given to suggest the widespread dissemination that would have been expected with a malignant lymphoma of seven years duration.

In our series of cases of malignant lymphomas we have observed 110 patients with tumours that we have grouped as lymphosarcoma. Of these, 39 had leukaemic changes in the peripheral blood. There was clinical evidence of involvement of the intracranial contents in one patient with lymphosarcoma without leukaemia and one patient with acute lymphatic leukaemia died of a cerebral haemorrhage. Post-mortem confirmation of the intracranial involvement was found in the first patient; in the second although there was no macroscopic evidence of infiltration, definite nodules of neoplastic tissue were found on microscopy.

---

### EXPLANATION OF PLATE.

FIG. 1.—Case 1 : Section of intracranial deposit removed at operation.
FIG. 2.—Case 1 : Section of intracranial deposit at autopsy.
FIG. 3.—Case 6 : Section of intracranial mass.
FIG. 4.—Case 7 : Section showing one of the intracerebral deposits.

1

2

3

4

John and Nabarro.

We cannot give any figures for the incidence of microscopic evidence of involvement of the brain or meninges in these cases because extensive histological study of these tissues was not undertaken.

*Case Reports.*

CASE 6.—In 1947, at the age of 46, the patient noted enlarged glands in his neck. Biopsy of one of these was reported as showing a malignant lymphoma. The glands responded to deep X-ray therapy given early in 1948, but shortly afterwards he developed pain behind the right eye, prominence of the right eye and double vision. He also complained of a purulent nasal discharge. X-rays showed infiltration of the frontal, ethmoidal and maxillary sinuses with partial destruction of the roof of the orbit, In June, 1948, he developed girdle pain and a complete paraplegia below the level of the 10th dorsal segment. On admission, he was found to have a right-sided proptosis, paralysis of the muscles of the right eye, movement of the palate to the right on phonation and signs of paraplegia. In addition, there were enlarged glands in the neck, axillae and groins, and a number of skin nodules. Following a course of nitrogen mustard injections the proptosis and glands regressed and the nasal discharge ceased, but there was no change in the paraplegia. His general condition continued to deteriorate rapidly and he died ten days after the completion of the course of nitrogen mustard.

At autopsy, the glands, including those of the mediastinum, were enlarged and infiltrated with yellow lymphoma. The brain was soft and oedematous. Anteriorly, apparently originating in the orbit, was a mass of greenish yellow necrotic tissue infiltrating the base of both frontal lobes. The tumour extended back to the optic chiasma, but the pituitary stalk was free. Both ventricles appeared normal and there was no evidence of raised intracranial pressure. Microscopically the tumour was extremely necrotic in places, but elsewhere the appearances were suggestive of a lymphoblastic lymphoma (Fig. 3).

*Comment.*—In this case there was a lymphoblastic lymphoma originating in the bones or tissue in the roof of the orbit and actually invading the brain at its base. The principal clinical evidence of intracranial involvement was a unilateral palatal weakness.

CASE 7.—The patient was a woman of 67, admitted to hospital with a history of general weakness, breathlessness and pallor for the preceding two months. On examination she had numerous petechiae, enlarged lymphatic glands, tonsils, liver and spleen. The blood count suggested a subacute lymphatic leukaemia and she improved little with blood transfussion and corticotrophin. Two weeks after admission, she became comatose with signs of cerebral haemorrhage and died within twenty-four hours.

At post-mortem, numerous petechiae were noted and infiltration of the lymph nodes, liver, spleen and bone marrow. There was a large left-sided cerebral haemorrhage with many small haemorrhages in the surrounding brain. Microscopically the haemorrhages were found to be associated with small leukaemic deposits (Fig. 4).

*Comment.*—This patient developed a cerebral haemorrhage as part of the haemorrhagic tendency of an acute or subacute leukaemia. It is presumably to be related to a thrombocytopenia although no platelet count was done. The nodular leukaemic infiltrations did not produce any clinical abnormality, although it is possible that they may have played some part in the development of the terminal haemorrhage by damage to the walls of the blood vessels.

### RETICULUM-CELL SARCOMA.

Primitive lymphomas in the so-called reticulum-cell sarcoma group present certain difficulties in recognition and in differentiation from other primitive mesenchymal tumours. We have restricted the term reticulum-cell sarcoma to multicentric tumours and have been only able to trace one possible case of this type with involvement of the intracranial contents. This is the second case reported by Abbott and Adson (1943).*

A number of writers have reported cases of primary reticulum-cell sarcoma of the brain. These cases have been reviewed by Troland, Sahyoun and Mandeville in 1950, who conclude that they should be grouped among the primary mesenchymal tumours of the brain and not as malignant lymphomata. In this group they also include cases of primary Hodgkin's sarcoma and primary lymphosarcoma of the brain.

### UNCLASSIFIED LYMPHOMA.

In addition to the cases already described, one of our patients with malignant lymphoma of controversial histological type had evidence, clinically, of invasion of the base of the skull and involvement of the cranial nerves. Unfortunately, the patient died at home and no autopsy was performed, so we are unable to give details of the anatomical or histological nature of the neoplasm.

### DISCUSSION.

In this report we have been concerned primarily with the clinical manifestations of intracranial malignant lymphoma. Our own cases have confirmed the general impression gained from the literature, that pathological evidence of such involvement is nearly as rare as clinical evidence although we cannot exclude the presence of microscopic deposits in all cases. The number of cases of Hodgkin's disease with intracranial deposits giving rise to symptoms and confirmed by post-mortem found in the literature is eight, and we are able to add one further case. Surgical or autopsy confirmation of such deposits is essential because of the evidence reported by Bateman, Squires and Thanhauser (1945) and Jackson and Parker (1945) of the occurrence of neurological symptoms in these patients without there being any corresponding neoplastic changes found at autopsy. We have reported one further case of this type (Case 2).

In most of the cases of intracranial Hodgkin's disease described in the literature the disease ran a rather rapid course and the neurological symptoms were a late development. Specific treatment has rarely been attempted although Fein and Newill's (1954) patient had irradiation and nitrogen mustard. In our patient there was some relief of symptoms with chemotherapy, but in view of the continued progress of the lesion and the patient's good general condition, surgery was undertaken. The results of this exploration were thought to have justified

* After this paper was submitted Allison and Gordon (1955, *Lancet*, ii, p. 120) reported a case of a reticulum cell sarcoma presenting with neurological symptoms. A cerebral deposit and meningeal infiltration were found at autopsy.

the course ; the patient lived a further fourteen months and the intracranial lesion gave him little further trouble. In cases of slowly progressing Hodgkin's disease, in which there is evidence of an intracranial deposit, the possibility of diagnostic studies for the localisation of such a lesion and its operative removal should be considered if the neurological symptoms develop before the disease enters its terminal stage. The types of disturbance that should suggest intracranial deposits are : evidence of raised intracranial tension, Jacksonian fits, cranial nerve palsies or other focal neurological signs.

In lymphosarcoma without leukaemic blood changes evidence of intracranial involvement is equally rare. We have collected five cases from the literature and added one of our own. In these cases the disease has nearly always been a rapidly-growing lymphoma in which the long-term response to treatment has been very disappointing. Four of the patients had cranial nerve palsies due either to nuclear involvement or infiltration of the nerve sheath. In one patient with Jacksonian epilepsy irradiation was claimed to have led to improvement, but the disease was widespread and rapidly proved fatal.

In patients with leukaemic blood changes in association with lymphosarcomatous infiltration of the lymphatic glands and bone marrow the reported incidence of neurological involvement is suprisingly high. The incidence of intracranial involvement is lower, and in many of these the pathological lesion is a cerebral haemorrhage. Whether this is due entirely to the low platelet count or whether some of the haemorrhages result from tumour nodules invading the vessel wall is uncertain. In our series of thirty-nine cases of lymphosarcoma with leukaemic changes in the peripheral blood, there was one patient who died of a cerebral haemorrhage.

Clinical evidence of intracranial involvement in generalised reticulum-cell sarcoma is apparently extremely rare and we have only been able to trace one possible case.* The condition of primary reticulum-cell sarcoma of the brain should probably not be included under the heading of malignant lymphoma.

### SUMMARY.

The literature relating to the clinical evidence of intracranial involvement in cases of malignant lymphoma has been reviewed.

Experience of this complication in a series of 347 cases of malignant lymphoma admitted to University College Hospital between 1946 and 1953 is reported. Ninety-three of the patients were examined at autopsy.

From the review of the literature and the findings in this series intracranial deposits of malignant lymphoma appear to be very uncommon.

One case of Hodgkin's disease with an intracranial deposit is reported. Partial removal was undertaken with considerable benefit to the patient. Other cases of Hodgkin's disease included one with symptoms suggesting intracranial deposits although none were found at post-mortem examination, and three with clinical evidence of intracranial manifestations who were not examined at autopsy. In one of these the symptoms were relieved by chemotherapy.

One case of lymphosarcoma invading the base of the brain from the bones of the skull is described. The high incidence of intracranial involvement reported in lymphatic leukaemia has not been confirmed in this series.

* See footnote to p. 398.

We wish to thank Dr. Gwen. Hilton, Director of the Radiotherapy Department at University College Hospital, to whom many of the patients were originally sent. We also would like to thank Dr. J. F. Stokes for permission to include Case 7, Professor G. R. Cameron for allowing us to refer to the autopsy reports, and Mr. E. Bligh for the photographs.

### REFERENCES.

ABBOTT, K. H. AND ADSON, A. W.—(1943) *Arch. Surg. Chicago*, **47**, 147.
ADAMS, R. D., DENNY-BROWN, D. AND FOLEY, J. M.—(1953) *Neurology*, **3**, 615.
BATEMAN, O. J., SQUIRES, G. AND THANHAUSER, S. J.—(1945) *Ann. intern. Med.*, **22**, 426.
COLRAT, A.—(1921) L'adénie éosinophilique prurigène, Thèse de Lyon.
DAVIDSON, C. AND MICHAELS, J. J.—(1930) *Arch. intern. Med.*, **45**, 908.
DIAMOND, I. B. (1934) *Arch. Neurol. Psychiat., Chicago*, **32**, 118.
FEIN, S. B. AND NEWILL, S. A.—(1954) *Amer. J. Med.*, **17**, 291.
GALL, E. A. AND MALLORY, T. B.—(1942) *Amer. J. Path.*, **18**, 381.
GINSBERG, S.—(1927) *Arch. intern. Med.*, **39**, 571.
GRAY, R. C., BAKER, A. B., COTRELL, L. AND KOGLAND, S.—(1941) *Int. Clin.*, **4**, 230.
HALPERN, L.—(1952) *Harefuah*, **41**, 153.
VON HECKER, H. AND FISCHER, W.—(1922) *Dtsch. med. Wschr.*, **48**, 482.
HOSTER, H. A., DRATMAN, M. B., CRAVER, L. F. AND ROLNICK, H. A.—(1948) *Cancer Res.*, **8**, 1.
JACKSON, H. AND PARKER, F.—(1945) *New Engl. J. Med.*, **233**, 369.
JOHNSSON, V.—(1931) *Hygiea*, **93**, 39.
KINNEY, T. D. AND ADAMS, R. D.—(1943) *Arch. Neurol. Psychiat., Chicago*, **50**, 552.
KOHUT, J.—(1946) *J. nerv. ment. Dis.*, **103**, 9.
LEIDLER, F. AND RUSSELL, W. O.—(1945) *Arch. Path.*, **40**, 14.
MARTIN, H. E. AND COURVILLE, C. B.—(1936) *Bull. Los Angeles neurol. Soc.*, **1**, 145.
MURCHISON, C.—(1869–70) *Trans. path. Soc. Lond.*, **31**, 372.
RADZINSKI, J. M. AND UZNANSKI, M. E.—(1944) *Illinois med. J.*, **85**, 87.
RIZZI, I.—(1938) *Riv. neurol.*, **2**, 377.
ROBB-SMITH, A. H. T.—(1938) *J. Path. Bact.*, **47**, 457.
SCHWAB, R. S. AND WEISS, S.—(1935) *Amer. J. med. Sci.*, **189**, 766.
SEREBRJANK, B.—(1933) *Dtsch. Z. Nervenheilk.*, **129**, 103.
SPARLING, H. J., ADAMS, R. D. AND PARKER, F.—(1947) *Medicine, Baltimore*, **26**, 285.
STERNBERG, C.—(1925) *Klin. Wschr.*, **4**, 529.
TROLAND, C. E., SAHYOUN, P. F. AND MANDEVILLE, F. B.—(1950) *J. Neuropath.*, **9**, 322.
VIETS, H. R. AND HUNTER, F. T.—(1933) *Arch. Neurol. Psychiat., Chicago*, **29**, 1246.
WELCH (1910) quoted by EUGENNIS, C.—(1929) Les Manifestations cérébro-medullaires de l'adénie éosinophilique prurigène, Thèse de Lyon.
WINKLEMAN, N. W. AND MOORE, T. M.—(1941) *Arch. Neurol. Psychiat., Chicago*, **45**, 304.
YOUNG, G. A., YOUNG, R. H. AND GYSIN, W. M.—(1945) *Neb. St. med. J.*, **30**, 434.